# Composite Membranes Based on MXene and Nanocellulose for Water Purification: Structure, Efficiency, and Future Prospects

**DOI:** 10.3390/membranes15100293

**Published:** 2025-09-26

**Authors:** Madina Suleimenova, Aidana Tabynbayeva, Kainaubek Toshtay, Zhandos Tauanov

**Affiliations:** 1 Faculty of Chemistry and Chemical Technology, Al-Farabi Kazakh National University, Al-Farabi Avenue 71, Almaty 050038, Kazakhstan; madina.suleimenova@kaznu.edu.kz (M.S.);; 2 Ecology Research Institute, Khoja Akhmet Yassawi International Kazakh-Turkish University, B. Sattarkhanov Avenue 29, Turkestan 161200, Kazakhstan

**Keywords:** MXene, nanocellulose, composite membranes, water purification, antifouling properties

## Abstract

The development of efficient and environmentally sustainable membrane materials is essential for advancing water purification technologies. This review examines composite membranes that combine the properties of MXene and nanocellulose, focusing on their structural features, functional characteristics, and potential advantages in water treatment applications. Nanocellulose provides a biodegradable, renewable matrix with abundant surface functional groups, while MXene offers high hydrophilicity, electrical conductivity, and adsorption capacity. Based on a critical evaluation of published studies, the review outlines various fabrication strategies, discusses key factors affecting membrane performance—including morphology, surface modification, and interfacial interactions—and highlights the synergistic effects between the two components. The article systematizes current approaches to designing MXene/nanocellulose membranes and establishes a foundation for future scientific and technological development in this field.

## 1. Introduction

Water pollution has emerged as one of the most critical environmental challenges of the 21st century, with profound implications for ecological integrity, public health, and sustainable development. The increasing discharge of pollutants from diverse anthropogenic sources—such as industrial effluents, agricultural runoff, mining activities, and domestic wastewater—has resulted in the contamination of aquatic ecosystems with complex mixtures of hazardous substances. These pollutants not only degrade water quality but also disrupt ecological balance, threaten biodiversity, and pose long-term risks to human health and socioeconomic stability. Among various pollutants, heavy metals—particularly mercury—are of significant concern due to their extreme toxicity, environmental persistence, and propensity for bioaccumulation in living organisms. According to the World Health Organization (WHO), the maximum permissible concentration of inorganic mercury in drinking water is 0.006 mg/L; however, several countries, including Australia, have adopted even stricter standards of 0.001 mg/L [1,2]. Major anthropogenic sources of mercury include industrial effluents, coal combustion, mining activities, and the improper disposal of mercury-containing waste. Within aquatic ecosystems, microbial processes transform inorganic mercury into methylmercury, a highly toxic form capable of both bioaccumulation and biomagnification in food webs, as demonstrated by recent environmental studies [3,4]. The Minamata tragedy remains emblematic of mercury’s devastating impacts: in 2023, a Japanese court recognized 128 additional victims and ordered compensation from both the government and Chisso Corporation [5]. Comparable incidents continue to emerge, such as in Brazil, where illegal gold mining has been linked to rising cases of mercury-related birth defects [6]. At the global level, regulatory efforts have been consolidated through the Minamata Convention on Mercury, signed by over 128 countries and entering into force in 2017, aiming to control and progressively reduce mercury emissions worldwide [5].

Composite membranes have emerged as a promising alternative to conventional water purification technologies for the removal of heavy metal ions, offering high selectivity, enhanced sorption capacity, and long-term durability. The functionalization of membranes with metal–organic frameworks (MOFs), such as UiO-66, combined with cellulose nanofibers, has demonstrated removal efficiencies exceeding 96% for Pb^2+^, Cu^2+^, and Cd^2+^ while maintaining stable water flux and mechanical integrity [7]. Moreover, covalently bonded cellulose-based active layers provide sorption capacities of up to 194 mg/g and ensure efficient performance even at low pollutant concentrations [8]. Similarly, graphene oxide (GO)-based hybrid membranes functionalized with bioactive quercetin have exhibited selective removal of Cr^6+^, As^3+^, Cd^2+^, and Pb^2+^ with efficiencies reaching 99.5%, coupled with high water permeability during prolonged operation [9]. Recent reviews highlight that these advanced composite materials enable the synergistic integration of membrane filtration and chemisorption processes while minimizing energy consumption and reducing secondary waste generation compared with conventional treatment methods.

In this context, MXene-based membranes (e.g., Ti_3_C_2_T_x_, Nb_2_C, and V_2_CT_x_) have emerged as highly efficient platforms for water purification owing to their unique two-dimensional lamellar architectures, tunable surface terminations (–OH, –O, and –F), and intrinsic hydrophilicity, which collectively enable rapid water transport and selective ion rejection. At high permeation rates (>200 L m^−2^ h^−1^ bar^−1^), Ti_3_C_2_T_x_ MXene membranes have demonstrated over 95% removal efficiencies for Pb^2+^ and Cr^6+^, whereas Nb_2_C membranes exhibit exceptional chemical stability under both acidic and alkaline conditions, rendering them suitable for industrial wastewater treatment. Furthermore, hybrid MXene–cellulose composites integrate the mechanical robustness of cellulose with the high surface area of MXenes, achieving effective removal of heavy metals and organic dyes without flux decline during long-term operation. The layered nanostructure enabling size-exclusion filtration, the abundance of oxygen-containing functional groups enhancing hydrophilicity, the versatile surface functionalization potential, and the compatibility with both polymeric and inorganic frameworks make graphene oxide (GO) a valuable component in advanced membrane design. Additionally, GO improves pollutant-binding affinity and fouling resistance by facilitating synergistic interactions with MXenes, cellulose nanofibers, and MOFs [10,11,12,13,14,15].

Modern composite membranes offer substantial advantages over conventional water purification technologies owing to their exceptional selectivity, chemical stability, and multifunctionality. For instance, GO membranes functionalized with onion extract (OE) and quercetin have demonstrated over 70% rejection of small inorganic ions (NaCl, Na_2_SO_4_, MgCl_2_, and MgSO_4_) in water desalination experiments [16]. Similarly, multilayer cellulose membranes incorporating covalently bonded active layers exhibit sorption capacities of up to 194 mg/g for Cu^2+^, Pb^2+^, and Cd^2+^ while retaining their performance characteristics after multiple regeneration cycles [10]. Anisotropic aerogels fabricated from cellulose nanofibrils modified with UiO-66 and EDTA achieve up to 98% removal efficiency for heavy metal ions at initial concentrations of 10 mg/L and maintain stable performance for at least five reuse cycles [17,18]. Moreover, recent designs such as GO@DA/PEI membranes attain both high water flux and ion selectivity through the synergistic interaction of polyethyleneimine and dopamine functional groups [19].

Representative examples of advanced membrane materials developed for the removal of heavy metal ions include the following:

Cellulose-based membranes: cellulose aerogels functionalized with EDTA, CNF–UiO-66 composites, and covalently bonded cellulose active layers.

GO-based membranes: GO–quercetin (GO/OE) and GO@DA/PEI hybrids, as well as reduced GO functionalized with amino groups.

MXene-based membranes: Ti_3_C_2_T_x_–cellulose nanofiber membranes, Nb_2_C MXene membranes, V_3_CT_x_ hybrid films, and MXene–GO nanocomposites.

Hybrid systems: GO–MOF membranes, MXene–MOF membranes, and MOF–cellulose membranes.

Despite significant advances in membrane technology, several challenges remain, including the need to improve stability under variable environmental conditions, enhance regeneration efficiency, and ensure cost-effective large-scale production. Notably, even at trace concentrations (<10 ppb), cellulose-based membranes functionalized with thiol or amine groups have demonstrated >98% removal efficiencies for Hg^2+^. Similarly, MXene-based membranes modified with cysteamine or dithiocarbamate groups have achieved >99% Hg^2+^ removal in both synthetic and real wastewater streams, exhibiting minimal performance decline over multiple regeneration cycles. Moreover, GO-based membranes incorporating sulfur-containing ligands display high selectivity toward Hg^2+^ over competing cations due to the strong Hg–S binding affinity [16,20]. This review aims to examine the potential of cellulose- and MXene-based composite membranes for the efficient removal of diverse water pollutants—including heavy metal ions (Hg^2+^, Pb^2+^, Cd^2+^, Cr^6+^, and As^3+^), organic contaminants, and other hazardous substances—while maintaining high water permeability and long-term operational stability.

## 2. MXene as a Promising Material for Membrane Technology

MXenes are a class of two-dimensional materials produced by selective etching of layered carbides, nitrides, or carbonitrides known as MAX phases (M_n+1_AX_n_, where M is a transition metal, A is an element from groups 13–15, and X is carbon and/or nitrogen). In contrast to graphene and transition metal dichalcogenides, whose layers are bound mainly by van der Waals forces, MXenes exhibit strong M–A bonds and versatile M–X bonds with metallic, covalent, or ionic character.

The resulting etched products are typically denoted as M_n+1_X_n_T_x_, where T represents surface functional groups such as –F, –OH, –Cl, and –O. These surface terminations critically influence the electronic conductivity, mechanical strength, hydrophilicity, chemical reactivity, and environmental stability of MXenes. Moreover, higher n values enhance thermodynamic stability by strengthening interlayer interactions and improving structural compatibility [21]

The key physicochemical properties of MXenes—including their structural characteristics, surface chemistry, mechanical strength, electrical conductivity, hydrophilicity, surface charge, and stability—are summarized in Table 1.

To date, more than 30 stoichiometric MXenes with diverse electronic, physical, and electrochemical properties have been experimentally synthesized, while over 100 additional compounds have been theoretically predicted (excluding surface terminations). Furthermore, solid-solution formation is possible at both the M and X sites, and the presence of multiple surface terminations—either single (e.g., O, Cl, F, S) or mixed (e.g., O/OH/F)—further broadens the compositional diversity of MXenes within the family of two-dimensional materials [23].

### MXene Synthesis Methods

MXenes are most commonly produced via top-down transformation of the parent MAX phases rather than through classical synthesis methods. In these approaches, the A-layer of the MAX phase is selectively removed to yield two-dimensional MXene structures. Among the widely employed techniques, chemical etching, electrochemical etching, and mechanochemical methods are most prominent [21,23,29,30]. More recently, promising bottom-up approaches—such as chemical vapor deposition (CVD)—have been reported [31], enabling the direct synthesis of MXenes from elemental precursors. A concise comparison of these methods is summarized in Table 2.

**Figure 1:**
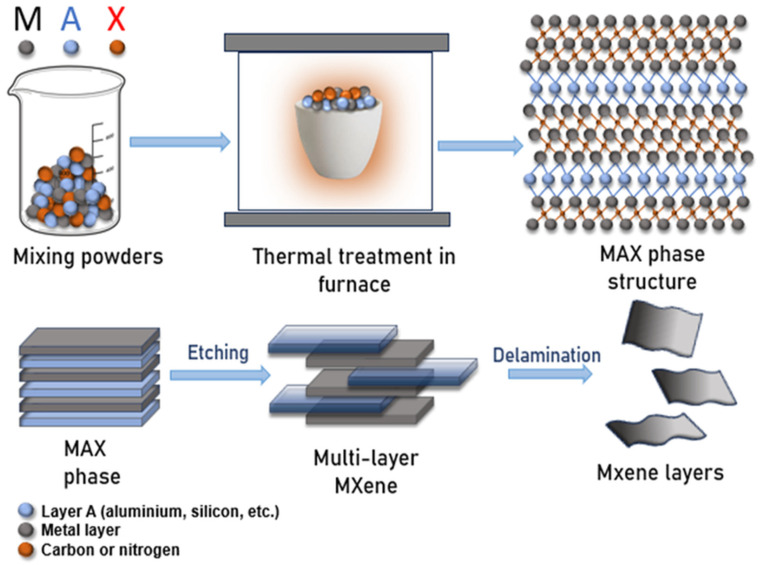
Schematic diagram of MXene synthesis.

The schematic diagram in Figure 1 illustrates the stepwise synthesis of the MAX phase. Initially, powders of the M, A, and X elements are combined in a predetermined stoichiometric ratio, where M (black) denotes a transition metal (e.g., titanium, vanadium, zirconium), A (blue) represents aluminum, silicon, or similar elements, and X (red) refers to carbon or nitrogen.

The resulting powder mixture is sintered at high temperatures under an inert atmosphere, leading to the formation of the crystalline MAX phase, in which transition metal layers (M) alternate with carbon or nitrogen layers (X), while A-layers occupy the interlayer spaces. This unique layered architecture makes the MAX phase an ideal precursor for the selective etching process used to produce MXenes.

Subsequently, the MAX phase undergoes chemical treatment with acids or alkalis (e.g., HF or LiF/HCl solutions), dissolving the A-layers and yielding a multilayer MXene structure. Further exfoliation techniques, such as ultrasonic treatment or mechanical dispersion, delaminate the multilayered material into individual MXene nanosheets, producing thin, two-dimensional flakes with large surface areas and high aspect ratios [37].

In this schematic, the overall process is intentionally simplified, with chemical bonds omitted to provide a clearer visualization of the sequential structural transformations. The diagram thus illustrates the crystal structure of the MAX phase, its synthesis route, and the subsequent conversion mechanism leading to MXene formation.

## 3. Classification, Properties, and Formation Mechanisms of Nanocellulose

### 3.1. Types of Nanocellulose

Nanocellulose possesses a high specific surface area, excellent mechanical strength, and inherent biocompatibility, making it highly suitable for advanced material design and water treatment applications. Based on morphology and production methods, nanocellulose is generally classified into three main types [38,39,40]:

- Bacterial nanocellulose (BNC): Synthesized by microorganisms such as Acetobacter xylinum, BNC is free from lignin and hemicellulose impurities, resulting in exceptional purity and porosity. These properties enable its use in biomedical applications and high-performance membranes [41].
- Crystalline nanocellulose (CNC): Consisting of rod-shaped nanoparticles produced via acid hydrolysis of crystalline cellulose, CNC exhibits high crystallinity, rigidity, and adsorption capacity. Its surface can be readily functionalized to enhance pollutant removal and mechanical reinforcement in composite membranes [42,43].
- Fibrillated nanocellulose (FNC): Comprising long, entangled fibrils with both amorphous and crystalline domains, FNC is typically obtained through mechanical or chemo-mechanical treatments. Its network structure improves interfacial adhesion and fouling resistance in hybrid membranes [40,44].

Since the surface chemistry of nanocellulose critically influences its compatibility with MXene-based membranes, the key functional groups, their chemical structures, and corresponding roles in membrane performance are summarized in Table 3.

### 3.2. Methods of Nanocellulose Production

Nanocellulose can be produced via two principal strategies: bottom-up and top-down approaches. Among these, top-down methods—such as mechanical, chemical, or chemo-mechanical treatments—are the most widely employed due to their efficiency and practicality in extracting nanocellulose from diverse biomass sources. The utilization of various lignocellulosic and agricultural residues not only enhances raw material availability but also aligns production with the principles of sustainability and environmental stewardship.

A comparative analysis of nanocellulose production techniques (Table 4) demonstrates that no single method provides a universal solution devoid of limitations. Each approach presents unique advantages and constraints, making the selection of an optimal production route highly context-dependent. For instance, alkaline and acid hydrolysis methods yield nanocellulose with high crystallinity and mechanical strength but require stringent control over reaction conditions, along with careful neutralization and disposal of chemical wastes. In contrast, biosynthetic routes exhibit excellent environmental compatibility but are hindered by slow production rates and elevated scaling costs. Mechanical methods circumvent the use of harsh chemicals, thereby minimizing environmental impact; however, they entail high energy consumption and frequently necessitate additional post-treatment steps to tailor the final properties of the nanocellulose.

In practice, researchers are increasingly adopting hybrid strategies, such as mechanical pretreatment followed by chemical or enzymatic processing. This sequential approach not only enhances yield and structural uniformity but also facilitates the fine-tuning of morphological and functional properties to meet the requirements of diverse applications, ranging from membrane technologies to biomedical systems and sustainable packaging materials. However, the economic feasibility of such integrated methods remains a critical challenge, particularly when scaling up from laboratory research to industrial production.

### 3.3. Mechanisms of Nanocellulose Formation

Intermolecular hydrogen bonding between cellulose chains imparts exceptional mechanical strength and structural stability, while nanocellulose exhibits pronounced axial stiffness. These hydrogen bonds govern key physicochemical properties of cellulose, including self-assembly behavior, crystallinity, molecular accessibility, and chemical reactivity. A higher density of hydrogen bonds promotes tightly packed molecular arrangements, fostering the development of crystalline domains, whereas regions with fewer bonds remain amorphous. Consequently, cellulose microfibrils comprise both crystalline and amorphous regions, with their relative proportions largely determined by the source of the plant material. For example, the crystallinity of cotton-derived cellulose can reach up to 80%, considerably higher than that of wood cellulose [68].

**Figure 2:**
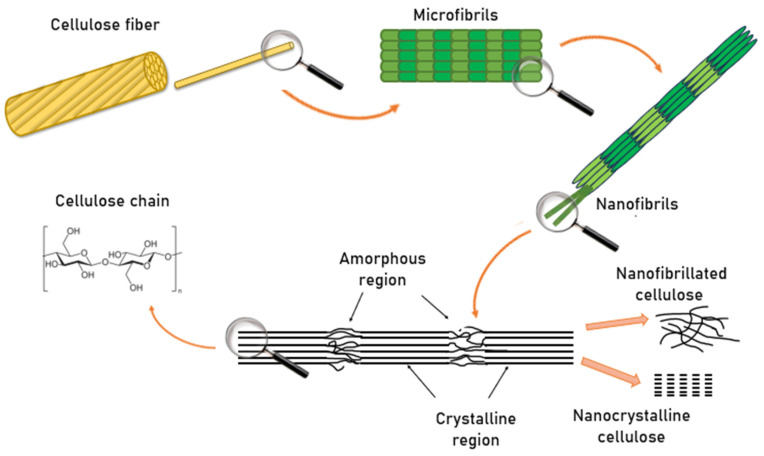
Mechanism of Nanocellulose Formation.

The conversion of cellulose into nanoscale structures proceeds through multiple sequential stages (Figure 2).

Initially, cellulose fibers exist as macroscopic structures composed of bundles of microfibrils tightly bound through hydrogen bonding and van der Waals interactions. When exposed to targeted mechanical, chemical, or enzymatic treatments, these hierarchical structures gradually disintegrate. In the first stage, the fibers are separated into microscale elements known as microfibrils. With further processing, these microfibrils are disintegrated into nanofibers or nanofibrils, which exhibit a high specific surface area and unique physicochemical properties.

At the molecular level, cellulose consists of long, linear polysaccharide chains of repeating β-D-glucopyranose units linked by β-1,4-glycosidic bonds. These chains form both crystalline and amorphous regions. The crystalline domains display dense molecular packing and a high degree of order, conferring exceptional mechanical strength and chemical stability. In contrast, the amorphous regions are characterized by a more disordered arrangement, rendering them more susceptible to chemical and enzymatic modifications.

Depending on processing conditions and the extent of structural disintegration, two principal types of nanocellulose can be distinguished. Nanofibrillated cellulose (CNF) consists of an interconnected network of elongated nanofibrils containing both crystalline and amorphous domains. Nanocrystalline cellulose (CNC), on the other hand, is obtained by selectively removing the amorphous regions, leaving behind highly ordered crystalline domains. Both CNF and CNC exhibit remarkable mechanical strength, biocompatibility, and renewability, making them highly attractive for applications in membrane technology, biomedicine, electronics, and sustainable packaging.

## 4. Composite Membranes

### 4.1. Classification of Composite Membranes

**Figure 3:**
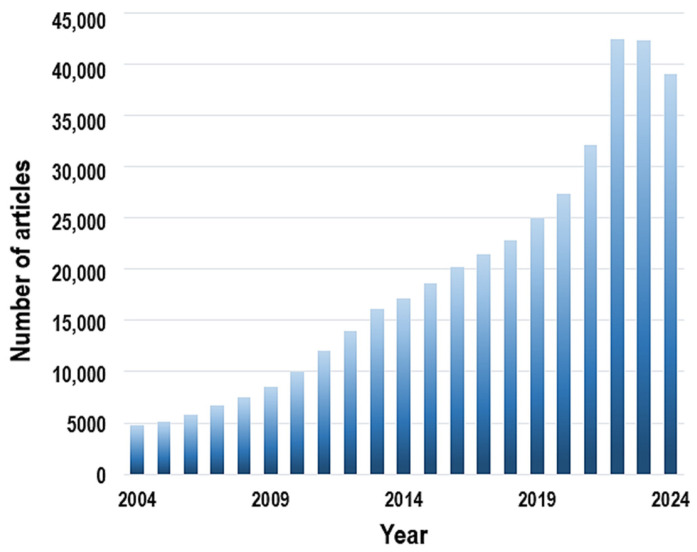
Annual growth of scientific publications on composite membranes for water purification.

Over the past 20 years, research on composite membranes for water purification has grown substantially. From 2004 to 2010, the number of publications increased gradually, but the pace accelerated with the advent of advanced nanomaterials such as TiO_2_, graphene, and MXenes, as well as hybrid structures including MOFs and polymer-based composites. After 2019, the research output rose sharply, with the number of studies surpassing 40,000 by 2022–2023, underscoring the escalating global demand for innovative and efficient water purification technologies (Figure 3).

The principal classes of composite membranes employed in separation processes are summarized in Table 5. This classification outlines the commonly used fillers and modifiers, their influence on membrane performance, and representative practical applications.

**Figure 4:**
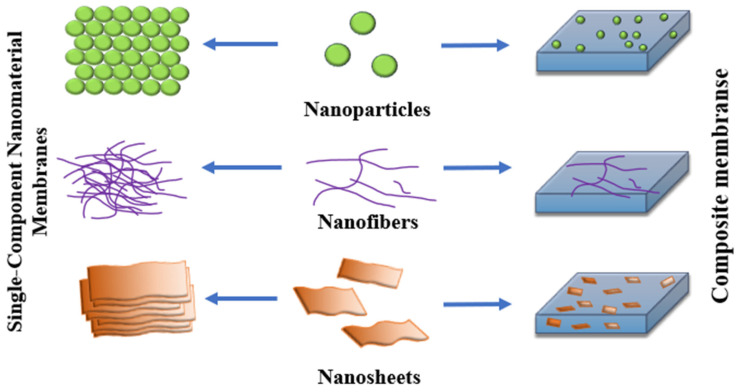
Integration of Nanostructures into Membrane Technologies.

Incorporating nanoparticles, nanofibers, and nanosheets into membrane matrices enables the creation of composite membranes with enhanced selectivity, permeability, and mechanical stability (Figure 4). This schematic depicts the transition from single-component nanomaterial membranes to multifunctional composites, highlighting the role of integrated nanostructures in improving water treatment and separation performance.

Over the past decade, composite membrane technology has witnessed remarkable progress, driven by the need for improved efficiency, selectivity, and operational stability. Advanced membranes—incorporating nanomaterials, hybrid and nanostructured architectures, biomimetic designs, and ionic liquid-based systems—are increasingly deployed in applications such as gas separation, water purification, and other separation processes. These innovations not only expand the functional capabilities of membranes but also pave the way for future breakthroughs in high-performance membrane technologies.

### 4.2. Materials Used in Composite Membranes

#### 4.2.1. Polymeric Matrices

Polymeric matrices commonly employed in composite membranes include polyamide, polysulfone (PSF), polyvinylidene fluoride (PVDF), chitosan, and polyacrylonitrile (PAN). These polymers confer excellent mechanical strength and chemical stability, making them suitable for diverse applications such as desalination, selective contaminant removal, and biomedical processes. For instance, polyamide and polyimide membranes are extensively utilized in desalination due to their high selectivity and resistance to fouling [78].

The incorporation of MXenes and polyaniline (PANI) into polysulfone membranes enhances both electrical conductivity and antifouling properties, improving performance in water purification and dye removal applications [79]. Similarly, hybrid MXene–nanocellulose membranes exhibit exceptional efficacy in heavy metal removal, dye adsorption, and oil–water separation, benefiting from the synergistic combination of MXene’s high surface area and tunable surface terminations with the flexibility and hydrophilicity of nanocellulose [80].

PVDF-based membranes are widely applied in membrane technologies. PVDF composites modified with metal–organic frameworks (e.g., MAF-4) demonstrate high water permeability and fouling resistance, rendering them promising for membrane distillation [81]. Additionally, PVDF membranes reinforced with anionic clay nanoparticles and carbon quantum dots show enhanced performance in heavy metal adsorption and detection [82].

Biopolymer-based membranes, such as chitosan, are gaining attention due to their biodegradability and high adsorption capacity. Chitosan membranes functionalized with titanium dioxide (TiO_2_) have demonstrated superior water purification performance [83]. PAN membranes modified with multilayer MXene/carbon nanotube nanostructures enable efficient removal of oils and dyes from industrial wastewater [84]. Furthermore, electrospun PVDF/PAN membranes have been employed as separators in sodium-ion batteries and pressure sensors, highlighting their versatility and expanding functional applications [85].

#### 4.2.2. Functional Fillers

Functional fillers are critical in tailoring the properties of composite membranes, enhancing mechanical strength, sorption capacity, and transport performance. Among these, carbon nanomaterials, metal oxides, zeolites, MXenes, and functionalized nanoparticles have received considerable attention.

In cellulose- and nanocellulose-based composites, fillers not only improve contaminant removal efficiency but also enable precise tuning of interlayer spacing and surface charge, facilitating selective separation of ions and organic pollutants. For example, the integration of Ti_3_C_2_T_x_ MXene nanosheets into nanocellulose matrices generates lamellar channels with controlled spacing, enhancing ion sieving while maintaining high water flux [86].

Carbon nanomaterials, including GO and CNTs, are widely employed to reinforce membrane matrices, improving selectivity, permeability, and fouling resistance [75]. Metal oxides such as TiO_2_, Fe_3_O_4_, and ZrO_2_ are valued for their photocatalytic and sorptive properties; TiO_2_ enhances resistance to biofouling [72], while Fe_3_O_4_ enables magnetic membranes for efficient heavy metal removal.

Zeolites and MXenes offer exceptional porosity and structural stability. MXene-based membranes, in particular, benefit from tunable interlayer spacing and strong compatibility with nanocellulose, achieving heavy metal rejection efficiencies exceeding 99% while preserving high permeability [87]. Functionalized nanoparticles, including silver, gold, and MOFs, impart antibacterial and catalytic activity, prolonging membrane lifespan and reducing maintenance requirements [88].

As summarized in Table 6, the incorporation of functional fillers—ranging from carbon nanomaterials (GO and CNTs) and metal oxides (TiO_2_, Fe_3_O_4_, and ZrO_2_) to zeolites, MXenes, and functional nanoparticles (Ag, Au, and MOFs)—significantly enhances membrane performance. These additives improve hydrophilicity, selectivity, electrical conductivity, and fouling resistance, while also conferring antibacterial properties, thereby expanding the application scope of composite membranes across water and air purification, gas and liquid separation, biomedicine, sensing technologies, and energy systems.

Thus, the incorporation of functional fillers in composite membranes offers significant opportunities to enhance their performance. The development of novel materials and integrated strategies enables the design of membranes with superior selectivity, permeability, and fouling resistance, rendering them highly promising for diverse applications, including water purification, gas separation, and biomedical technologies.

#### 4.2.3. Functional Groups for the Selective Binding of Hg^2+^ Ions and Other Heavy Metals

Thiol, carboxyl, and amino groups form strong complexes with metal ions, thereby significantly enhancing sorption efficiency. Their high chemical reactivity makes them particularly effective for the removal of metal ions from aqueous solutions. Numerous studies have demonstrated that these functional groups exhibit high selectivity toward Pb^2+^, Cd^2+^, Ni^2+^, and Hg^2+^ ions. For example, magnetic nanosorbents functionalized with carboxyl and thiol groups can simultaneously and efficiently remove multiple metal ions [97].

Activated carbon modified with amino and thiol groups has been shown to achieve near-complete removal of Pb^2+^ and Hg^2+^ ions. These materials display high sorption rates, with the adsorption process governed by a combination of chemical complexation and ion-exchange mechanisms. Furthermore, these functionalized carbon sorbents are reusable and can be regenerated over multiple cycles [98].

Chitosan and its derivatives are also widely employed for heavy metal binding due to the presence of amino and hydroxyl groups within their structure. These biopolymers serve as eco-friendly biosorbents with high adsorption capacities [99]. Collectively, these findings underscore the ongoing relevance and importance of developing functionalized sorbents for highly efficient removal of heavy metal ions from water.

## 5. Efficiency and Selectivity of Composite Membranes in Water Treatment

Various characteristics such as water absorption, porosity, selectivity, antifouling properties, and thermal stability are used to evaluate the properties of membranes.

### 5.1. Water Absorption of Membranes

Water absorption of membranes is an important parameter that determines their hydrophilicity and ability to retain moisture. This parameter is calculated using the following formula:

$$\%\;\mathrm{Water}\;\mathrm{Absorption}=\left(\frac{W_{w}{-\;W}_{d}}{W_{d}}\right)\times 100$$

Here, *W_w_* denotes the mass of the wet membrane, the weight after soaking and removal of excess liquid, and *W_d_* denotes the mass of the dry membrane, the weight after complete drying to constant mass under specified conditions.

### 5.2. Membrane Porosity

Membrane porosity determines its ability to transmit liquids and gases, and also affects its selectivity and mechanical strength. It is calculated using the following formula:

Here, ρ is the density of water (0.998 g/cm^3^), *l* is the membrane thickness (cm), *A* is the membrane area (cm^2^).

$$\in (\%)=\left(\frac{W_{w}{-W}_{d}}{Al\rho }\right)\times 100$$

### 5.3. Antifouling Properties of Membranes

The antifouling properties of membranes determine their resistance to fouling and their ability to recover the flow after cleaning:

Here, $J_{f_{1}}$ is the initial pure water flux,${\;\;J}_{b}$ is the flux after filtering the foulant, and $J_{f_{2}}$ is the flux after cleaning the membrane.

Flux Recovery Ratio (*FRR*):

$$FRR\left(\%\right)=\left(\frac{J_{f_{2}}}{J_{f_{1}}}\right)\times 100$$

Reversible Fouling (*R_r_*)

$$R_{r}\left(\%\right)=\left(\frac{J_{f_{2}}{-J}_{b}}{J_{f_{1}}}\right)\times 100$$

Irreversible Fouling (*R_ir_*):

$$R_{ir}\left(\%\right)=\left(\frac{J_{f_{1}}{-\;J}_{f_{2}}}{J_{f_{1}}}\right)\times 100$$

Total Fouling Ratio (*R_t_*):

$$R_{t}\left(\%\right)=\left(\frac{J_{f_{1}}{-\;J}_{b}}{J_{f_{1}}}\right)\times 100$$

### 5.4. Selectivity of Composite Membranes

The separation efficiency of composite membranes can be evaluated using standard methods applied in membrane filtration. One such method includes calculating the rejection coefficient (*R*) and permeate flux (*J_p_*).

The rejection coefficient (*R*) determines how effectively the membrane retains organic compounds, dyes, or heavy metal ions. This indicator is calculated based on the concentrations of the substance in the feed solution (*C_f_*) and in the permeate (*C_p_*), quantitatively describing the membrane’s separation capability:

$$R=\left(1-\frac{C_{p}}{C_{f}}\right)\times 100\%$$

Permeate flux (*J_p_*) characterizes the permeability properties of the membrane and is determined by measuring the volume of liquid that passes through its surface over a certain period of time:

$$J_{p}=\frac{V}{A\times ∆t}$$

Here, *V* is the volume of the permeate, *A* is the effective area of the membrane, and ∆*t* is the testing time.

Such methods are widely used in the study of membrane technologies and allow for comparison of the effectiveness of different membranes in removing contaminants from water [100].

In addition to quantitative performance metrics such as water flux, porosity, antifouling resistance, and dye/salt selectivity, it is crucial to understand the underlying mechanisms that enable the superior function of MXene/nanocellulose composite membranes. Incorporation of Ti_3_C_2_T_x_ (MXene) into the polyamide active layer via interfacial polymerization induces a distinctive bubble-like surface morphology, substantially increasing the effective permeable area. This structural modification enhances water flux to approximately 45 L·m^−2^·h^−1^·bar^−1^—a 1.58-fold improvement over pristine polyamide membranes—while maintaining ultrahigh dye-to-salt separation coefficients (~820) [101]. MXene’s exceptional hydrophilicity and electrical conductivity further promote the formation of a stable hydration layer, which mitigates fouling. In ultrafiltration experiments, applying a negative electrochemical bias (2 V) increased permeate flux by 1.6-fold and improved total organic carbon removal from 60.7% to 71.2%, attributed to intensified electrostatic repulsion of foulants [102].

Chemical stability is another key advantage of MXene. Its robust surface chemistry enhances resistance to harsh conditions such as chlorination and extreme pH values. Thin-film nanocomposite (TFN) membranes reinforced with MXene retain structural integrity and filtration performance even under aggressive chemical environments [103]. For example, Natu et al. reported that aqueous Ti_3_C_2_T_x_ suspensions remain stable at neutral pH but exhibit significant aggregation at pH 5 and 10, indicating sensitivity to extreme acidity or alkalinity [104]. MXene-embedded nanofiltration membranes also maintained over 98% Na_2_SO_4_ rejection after 105 days of continuous water immersion, confirming long-term filtration stability [105].

In MXene–nanocellulose composite membranes, nanocellulose acts as a stabilizing matrix, preventing MXene layer aggregation and sealing defects via strong interfacial interactions. This synergy enhances mechanical strength, selectivity, and long-term antifouling performance [106]. By elucidating these mechanisms, it becomes evident that the integration of MXene and nanocellulose can simultaneously improve water permeability, selective separation, chemical robustness, and fouling resistance, making these composites highly promising for next-generation sustainable water purification technologies.

As summarized in Table 5, composite membranes based on different polymer matrices and functional fillers exhibit substantial variation in water permeability, flux recovery ratio (FRR), and contaminant removal efficiency. PSF/MXene and PES/Zwitterion-MXene membranes achieve high permeability (450–480 L·m^−2^·h^−1^·bar^−1^) while maintaining notable FRR (up to 94%) and BSA removal efficiency (up to 90%). MXene/ceramic substrates and PAN/CS/Fe_3_O_4_ composites excel in humic acid removal (86.5–96.5%), albeit with lower permeability. PAN/ZnO-NP membranes demonstrate high selectivity for Mn^7+^ ions (96.2%) with acceptable flux (136.3 L·m^−2^·h^−1^·bar^−1^), while mesoporous silica-based PES membranes achieve 91–94% removal of Cd^2+^ and Zn^2+^.

MXene–cellulose composites further enhance membrane adaptability. MXene/Ag_2_S–cellulose membranes remove methylene blue with 93.3% efficiency, and Ti_3_C_2_T_x_–cellulose composites achieve 99.5% removal of BSA and methyl green while maintaining an FRR of 67.3%. Ti_3_C_2_T_x_/cellulose acetate membranes exhibit exceptional antibacterial activity against *Bacillus subtilis* and *E. coli*, with permeability of 256.85 L·m^−2^·h^−1^·bar^−1^, FRR of 98%, and superior pollutant removal capacity. These results demonstrate that combining cellulose’s hydrophilicity and mechanical strength with MXene’s tunable surface chemistry and high conductivity produces membranes with excellent permeability, fouling resistance, and selective pollutant removal [107,108,109,110,111,112]. A comprehensive performance comparison of composite membranes for various contaminants is provided in Table 7.

## 6. Composite Membranes Based on MXene and Nanocellulose

### 6.1. Membrane Fabrication Methods

#### 6.1.1. Vacuum Filtration

Vacuum filtration followed by hot pressing is a rapid, straightforward, and accessible technique for fabricating layered nanocellulose membrane filters. The thickness and pore size of these membranes can be tuned by adjusting the concentration and volume of the nanocellulose suspension [119]. The effectiveness of this approach stems from its multifunctional nature: during vacuum filtration, nanocellulose fibers are densely packed, while subsequent hot pressing strengthens inter-fiber bonding and enhances mechanical integrity. Consequently, the resulting membranes can be tailored for diverse applications, including water purification, biomedical coatings, and catalytic processes.

#### 6.1.2. Evaporation and Self-Supporting Coating Deposition

**Figure 5:**
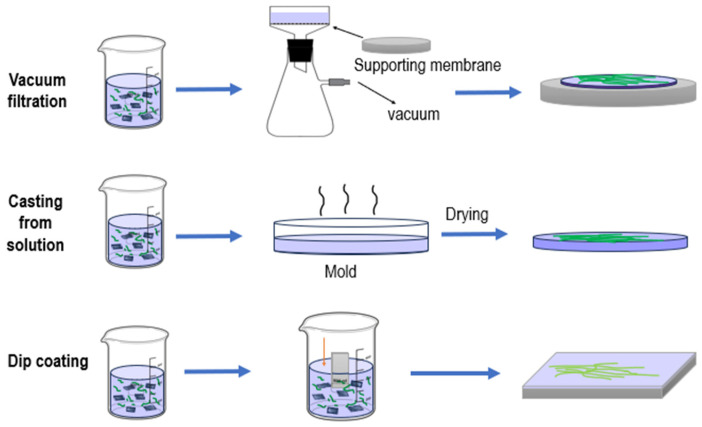
Membrane fabrication methods.

Self-supporting nanocellulose membranes are typically prepared by evaporating a dilute suspension in a Petri dish. To prevent fiber agglomeration, the dispersion concentration is maintained at a minimum, generally not exceeding 1 wt%, depending on fiber diameter and surface chemistry [119]. Although this method is simple, it has certain limitations: the evaporation process is time-consuming, reducing potential efficiency for industrial-scale production, and the uniformity of the resulting membranes depends on the initial dispersion state and evaporation rate. Nonetheless, this approach is effective for producing thin, highly transparent membranes, making it particularly suitable for optical devices and sensor coatings. Figure 5 (Membrane Fabrication Methods) provides a schematic overview of the main fabrication techniques discussed, including vacuum filtration, solution casting, and dip coating, with the sequence of operations illustrated for each method.

### 6.2. The Role of MXene and Nanocellulose in Composite Membranes

In recent years, composite membranes combining MXene and nanocellulose have attracted considerable attention due to the synergistic properties of these materials. Nanocellulose, in the form of CNF or CNC, provides mechanical strength, a porous structure, and hydrophilicity, whereas MXene nanosheets contribute electroactivity, functional versatility, and additional pathways for water and ion transport.

Recent studies report the fabrication of composite membranes integrating porous MXene layers (PMXene), engineered via etching to introduce nanopores, with cellulose nanofibrils (CNF). This strategy generates a stable three-dimensional framework with nanoscale channels, enhancing cationic conductivity. Consequently, both ion flux and selectivity are significantly improved, highlighting the potential of these membranes for applications such as osmotic energy generation [120].

#### TFN Membranes Based on Nanocellulose

Modern thin-film nanocomposite (TFN) membrane technologies increasingly leverage nanoparticles to enhance performance. For instance, functionalized cellulose nanocrystals (CNC), such as acetylated (ACNC) or L-cysteine-modified (CysCNC) particles, are incorporated into the organic phase prior to polymerization, simultaneously increasing water permeability and promoting efficient removal of salts and heavy metals [121].

MXene nanosheets also exert a profound influence on TFN membranes. Incorporation of Ti_3_C_2_T_x_ into the polyamide selective layer (0–0.02 wt%) reduces layer thickness and surface roughness, enhances hydrophilicity, and increases water flux to 2.3–2.5 L·m^−2^·h^−1^·bar^−1^ compared to 1.7 L·m^−2^·h^−1^·bar^−1^ for the pristine membrane, while maintaining high salt rejection (97.9–98.5%) and improving fouling resistance (flux decline reduced from 22.7% to 11.1%) [122]. Similarly, embedding MXene nanoparticles in thin-film composite forward osmosis (TFC-FO) membranes mitigates internal concentration polarization, decreasing the water contact angle by ~16%, enhancing cross-linking (O/N ratio decreased by 13%), and improving hydrophilicity and mass transport. These modifications yield ~80% higher water flux and improved reverse solute flux [123].

In these hybrid systems, MXene nanosheets provide tunable interlayer spacing and functional groups for selective heavy metal binding, while nanocellulose offers a flexible, hydrophilic scaffold that enhances flux and mechanical integrity. The hydroxyl groups of cellulose confer high hydrophilicity, which promotes sorption capacity but can also induce excessive swelling and reduce mechanical stability. Coupling nanocellulose with hydrophobic components such as MXene or chemically modified polymers allows fine-tuning of water permeability and mechanical performance, producing membranes with superior selectivity and flux for water purification.

The synergy between MXene and nanocellulose is notable: MXene compensates for the relatively low electrical conductivity of nanocellulose, while nanocellulose enhances mechanical stability, mitigates MXene aggregation, and reduces brittleness. Consequently, these hybrid membranes exhibit high flux, selective removal of heavy metals, dyes, and other pollutants, and multifunctionality suitable for applications in flexible electronics, supercapacitors, sensors, and barrier coatings. Additionally, variations in nanocellulose production methods (chemical, mechanical, or biological) allow tuning of its dimensions and properties, enabling MXene-based composites to be tailored for specific applications.

MXene–cellulose membranes have demonstrated exceptional efficacy in water treatment. For example, MXene/Ag_2_S–cellulose composites effectively remove dyes, while Ti_3_C_2_T_x_–cellulose structures efficiently adsorb proteins such as BSA and organic pollutants like malachite green. MXene–cellulose acetate membranes are particularly effective for disinfection-oriented filtration, exhibiting strong antibacterial activity against *Bacillus subtilis* and *E. coli*. These examples illustrate how the functional surfaces of MXene and the robust, hydrophilic network of nanocellulose can be combined to create adaptable membranes capable of targeting a broad spectrum of water contaminants.

**Figure 6:**
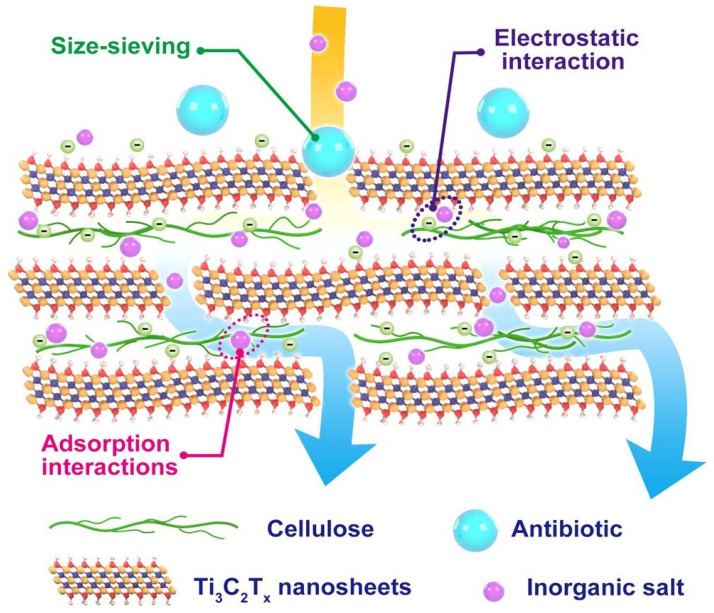
Schematic illustration of separation mechanism in Ti_3_C_2_T_x_/cellulose membranes. Reproduced with permission from Ref. [86].

As illustrated in Figure 6, Ti_3_C_2_T_x_/cellulose composite membranes employ multiple separation mechanisms: size exclusion governs the rejection of larger molecules, electrostatic interactions with functional groups on MXene nanosheets facilitate removal of charged species, and adsorption onto cellulose chains and MXene surfaces enables retention of organic molecules, including antibiotics. This synergistic interplay markedly enhances the simultaneous removal of both organic and inorganic pollutants, highlighting the promise of MXene–nanocellulose composites for advanced water purification, while retaining multifunctionality for potential applications in energy storage, sensing, and barrier technologies.

## 7. Prospects and Future Directions

### 7.1. Development of Self-Healing and Self-Cleaning Membranes

The pursuit of advanced membrane materials is increasingly focused on integrating multifunctional properties, particularly self-healing and self-cleaning abilities, to improve performance and durability. For instance, Xia et al. [124] developed a bioinspired supramolecular fibrous membrane that combines high mechanical strength with autonomous self-repair and self-cleaning, making it highly effective for water purification. Parallel efforts explore dynamic hybrid membranes designed to respond adaptively to environmental conditions, thereby conferring superior antifouling capacity and extending service life [125]. Enhancing these properties, research on MXene-based TFN membranes indicates that hydrophilic surface terminations (–OH, –F) critically enhance self-cleaning by promoting hydration layer formation and reducing foulant adhesion [126]. This understanding provides a valuable mechanistic framework for designing next-generation composites, including MXene-CNF systems.

### 7.2. Development of Environmentally Friendly Biodegradable Membranes

Nanofibrous membranes fabricated from cellulose acetate (CA) are attracting considerable research interest as biodegradable and eco-friendly alternatives to conventional polymer membranes. The electrospinning technique enables their fabrication with green solvents, such as water and ethanol, offering a sustainable synthesis route devoid of toxic reagents. A key advantage of this method is the facile tunability of fiber morphology and membrane porosity through processing parameters, facilitating optimization for specific applications in filtration, biomedicine, and environmental remediation [127]. Nonetheless, electrospun CA represents just one avenue for developing biodegradable membranes. A broad spectrum of modified and hybrid architectures based on natural polymers is under active investigation, each presenting a distinct profile of advantages and limitations regarding mechanical strength, permeability, porosity, and environmental sustainability.

Among these, nanocellulose stands out as a renewable platform characterized by high biocompatibility and a demonstrated capacity to stabilize MXene nanosheets against aggregation in composite structures [126]. Although cellulose-MXene composite membranes exhibit significant promise for advanced water filtration, their widespread application is hindered by several material-specific challenges. The operational lifetime and functional performance are compromised by the susceptibility of MXenes (e.g., Ti_3_C_2_T_x_) to oxidative degradation in aqueous environments. Conversely, the high hydrophilicity of nanocellulose, while beneficial for water permeability, often leads to excessive swelling, which can cause structural deformation and a consequent loss of mechanical stability. Achieving a homogeneous dispersion of MXene within the cellulose matrix is also critical, as nanosheet aggregation reduces the active surface area and diminishes filtration efficiency. Furthermore, ensuring long-term stability under dynamic aqueous conditions—including variations in pH, salinity, and temperature—is imperative for reliable large-scale deployment.

To address these challenges and enhance environmental durability, future research should focus on strategic material modifications. Techniques such as polymer grafting, crosslinking, or hybridization with hydrophobic protective layers can mitigate oxidation and swelling. Advanced fabrication methods, including layer-by-layer assembly and 3D printing, offer pathways for precise structural control without compromising scalability. To augment functionality, incorporating antimicrobial agents could enhance pathogen removal, while the functionalization of MXene and nanocellulose with specific chelating groups could enable the selective removal of toxic heavy metal ions like Hg^2+^ and Pb^2+^. By synergistically combining the structural robustness of nanocellulose with the tunable surface chemistry and conductivity of MXene, the development of multifunctional, stable, and sustainable membranes is an attainable goal. These advancements are crucial for translating these promising composite systems into practical, next-generation water treatment technologies.

### 7.3. Enhancing Functionality Through Molecular Design

To advance membrane performance beyond the conventional permeability-selectivity trade-off, the field is increasingly leveraging molecular-level modeling and precision material design. Wang et al. demonstrated the potential of this approach by developing organic molecular sieve-like membranes capable of separating complex mixtures with high selectivity [128]. Complementarily, molecular modeling has proven effective for optimizing the architecture of thin-film nanocomposite (TFN) membranes for reverse osmosis, leading to significant performance enhancements [129].

Recent investigations into MXene-based TFN membranes provide compelling quantitative evidence for the success of such molecular design strategies. For instance:

The interfacial incorporation of TA-Fe(III)-functionalized Ti_3_C_2_T_x_ nanosheets into the polyamide layer yielded a TFN membrane with a water permeability of approximately 12.17 L·m^−2^·h^−1^·bar^−1^—nearly double that of the pristine membrane—while maintaining a high Na_2_SO_4_ rejection rate (>97%). This configuration also conferred superior antifouling resistance and mechanical stability, which are critical for practical saline water treatment [130].

Utilizing MXene nanosheets as an interlayer template resulted in a dramatic ≈ 4.5-fold increase in permeance, from 10.2 to 45.7 L·m^−2^·h^−1^·bar^−1^, without compromising sodium sulfate rejection (>96.0%) [131].

Systematic analysis revealed that MXene incorporation creates nanovoids adjacent to the nanosheets, which function as efficient water nanochannels. This design yielded a 2.8-fold enhancement in permeance alongside a Na_2_SO_4_ rejection of 96.4%, with the channels demonstrating stability under sustained operational pressure [132].

Collectively, these studies underscore the transformative potential of rational nanomaterial integration in crafting high-performance separation membranes.

### 7.4. Use of Composite Membranes in Large-Scale Water Treatment Systems

Composite membranes are progressively being implemented in industrial and municipal water treatment systems, where their superior performance and enhanced fouling resistance are increasingly recognized [133]. Complementing this trend, Issaoui et al. have articulated the critical role of advanced membrane technologies in the sustainable management of water resources, delineating both persistent challenges and prospective solutions within the field [134]. Consequently, the continued integration and scaling of high-performance composite membranes represent a pivotal direction for the evolution of filtration technology.

In summary, the ongoing development of membrane technologies is fundamentally directed at overcoming critical limitations associated with durability, environmental impact, and functional efficacy. Contemporary research demonstrates that strategic approaches—including the incorporation of self-healing mechanisms, biodegradable polymers, molecular-level design, and composite architectures—significantly enhance performance metrics and broaden application scope. To propel the field forward, future endeavors must concentrate on the seamless integration of these innovative materials with scalable fabrication processes, ultimately ensuring robust efficiency and long-term stability under real-world operational conditions.

## 8. Conclusions

This review comprehensively examines the current landscape and future prospects of composite membranes incorporating nanocellulose and MXene, a class of materials garnering significant interest for advanced water purification. Nanocellulose offers an ideal sustainable matrix, characterized by its biocompatibility, biodegradability, exceptional mechanical strength, and high specific surface area. MXene, conversely, contributes high electrical conductivity, inherent hydrophilicity, and tunable surface chemistry, which collectively enhance membrane permeability and selectivity.

The synergy between these distinct nanomaterials facilitates the design of next-generation hybrid membranes with exemplary performance in demanding applications such as filtration, wastewater reclamation, and desalination. Empirical studies confirm that strategic structural design and surface functionalization of nanocellulose–MXene composites are pivotal for augmenting their mechanical robustness, chemical resistance, and operational longevity.

Notwithstanding this promising potential, key challenges persist, particularly concerning the scalability of manufacturing, the long-term stability of functional properties under operational stress, and the customization of membranes for specific aqueous environments. To address these hurdles, future research must prioritize the optimization of composite formulations, the development of scalable and sustainable fabrication protocols, and a fundamental elucidation of the nanoscale interaction mechanisms between the components. Such endeavors are crucial for translating these advanced materials into efficient and sustainable solutions for global water treatment challenges.

## Figures and Tables

**Table 1:** Physicochemical Properties of Mxene.

Physicochemical Property	Characteristics	Ref.
Structure	Layered, 2D nanolayers with high specific surface area	[22]
Surface groups	Functionalized with –OH, –O, –F groups depending on the synthesis method	[23]
Mechanical strength	Young’s modulus for the Ti_3_C_2_T_x_ monolayer is 0.484 ± 0.013 TPa, close to the theoretical value (0.502 TPa)	[24]
Electrical conductivity	Electrical conductivity of the Ti_3_C_2_T_x_ monolayer reaches up to 11,000 S·cm^−1^, indicating high current conductivity	[25]
Hydrophilicity	Exhibits high hydrophilicity due to terminal groups	[26]
Surface charge	Negative surface charge (depends on pH and terminal groups)	[27]
Stability	Prone to oxidation in aqueous media	[28]

**Table 2:** Comparative overview of MXene synthesis methods and their impact on membrane properties.

Method	Conditions/Reagents	Advantages	Limitations	Effect on Membrane Properties	Ref.
Chemical etching	HF, LiF/HCl; room temp. −60 °C	Produces multilayer MXene; relatively simple	Use of toxic HF, structural defects	Enhances hydrophilicity, dispersibility; improves water flux, selectivity; reduces fouling	[21,29,30,32]
Electro- chemical etching	Electrolytes (NH_4_Cl, HCl, NaClO_4_); applied voltage	Safer than HF; scalable; controllable	Requires precise voltage/current control	Generates defect-rich MXene; increases active sites; improves ion transport and rejection	[33,34,35]
Mechano- chemical treatment	High-energy ball milling with additives (salts, polymers)	HF-free; low cost; environmentally friendly	Non-uniform flakes, possible contamination	Improves polymer compatibility; enhances stability; antifouling	[35,36]
CVD growth	CH_4_, TiCl_4_, H_2_, Ar; 800–1000 °C	High-quality, uniform films	High temperature, expensive equipment	Stable MXene films; increases selectivity and conductivity	[31]

**Table 3:** Functional groups of nanocellulose.

Functional Group	Chem. Structure	Properties	Effect on Membrane Characteristics	Ref.
Hydroxyl	–OH	High reactivity, ability to form hydrogen bonds	Enhances membrane hydrophilicity, improves interaction with other materials	[45]
Carboxyl	–COOH	High acidity, ion exchange capability	Improves selective retention of heavy metal ions, increases surface charge	[46]
Sulfate	–OSO_3_H	Strong acidity, high solubility in water	Improves nanocellulose water solubility, enhances suspension stability, affects electrostatic properties	[47]
Aldehyde	–CHO	High reactivity, prone to oxidation	Enables further chemical modification of membranes, enhances bonding with other polymers	[48]

**Table 4:** Comparative analysis of nanocellulose production methods.

Method	Type of NC	Principle	Advantages	Disadvantages	Membrane-Related Properties	Ref.
Alkaline hydrolysis	CNF	Alkaline medium removes lignin and hemicellulose	Improved mechanical properties	Not eco-friendly, possible damage	Reveals OH groups, hydrophilicity	[49,50]
Acid hydrolysis	CNC	H_2_SO_4_ or HCl removes amorphous regions	High crystallinity, stable	Aggressive acids, neutralization needed	Compact CNC, strength, permeability	[51,52]
Bacterial synthesis	BNC	Fermentation by Komagataeibacter	Eco- friendly, pure cellulose	Slow (7–14 d), costly	3D networks, tunable porosity, high flux	[53,54]
Enzymatic hydrolysis	CNC, CNF	Cellulolytic enzymes hydrolyze cellulose	Eco-friendly, mild, high quality	Expensive enzymes, slow	Stable cellulose, low surface charge	[55,56,57,58]
Mechanical grinding	CNF	Physical fiber size reduction	Simple, no chemicals	High energy/equipment cost	Surface area, needs pretreatments	[59,60]
Ultrasonic treatment	CNF	Sound waves break fibers	Eco-friendly, good dispersion	Costly, fiber damage	Dispersion, better pores	[61,62,63]
Homogenization	CNF	High-pressure mechanical dispersion	Uniform fibers, stable	Pretreatment and high energy	Dense networks, good rejection	[64,65]
TEMPO oxidation	CNF	C6–OH → COOH (TEMPO/NaBr/NaClO)	Controlled, high dispersibility	Expensive reagents, env. issues	Dispersion, carboxyl groups	[66,67]

**Table 5:** Classification of Composite Membranes for Water Purification.

Type of Composite Membrane	Fillers/Modifiers	Key Properties/Applications	Ref.
Polymer composite membranes with functionalized nanomaterials	MXene, TiO_2_ nanoparticles	Improved hydrophilicity, ion selectivity, mechanical and thermal stability, suitable for water purification and gas separation	[69,70]
Hybrid membranes with inorganic fillers	MOFs, zeolites, TiO_2_/ZrO_2_	High selectivity, pressure resistance, effective for gas and liquid separation	[71,72,73]
Nanostructured membranes	GO, CNTs	Controlled pore size, efficient ion transport, ultrapermeability with low energy input	[74,75]
Biomimetic membranes	Aquaporins, bio-inspired coatings	High permeability, fouling resistance, natural-like filtration mechanisms	[76]
Ionic liquid-based membranes	Ionic liquids, IL–chitosan	Antifouling, high selectivity for ions and organic molecules, chemical resistance	[77]

**Table 6:** Effect of Various Functional Fillers on Membrane Properties.

Filler Type	Main Properties	Effect on Membrane	Application Area	Ref.
Carbon nanomaterials (GO, CNTs)	High mechanical strength, electrical conductivity, large surface area	Improves hydrophilicity and membrane selectivity	Water purification, gas separation, biomedicine	[89,90]
Metal oxides (TiO_2_, Fe_3_O_4_, ZrO_2_)	Photocatalysis, magnetic properties, high chemical stability	Adds antibacterial properties, improves heavy metal removal	Water and air purification, catalysis	[91]
Zeolites	High porosity, high selectivity	Enhances sorption of pollutants	Gas and liquid separation, water purification	[92,93]
MXene	High conductivity, unique mechanical properties	Enables fabrication of highly conductive membranes	Smart membrane systems, energy storage	[94]
Functional nanoparticles (Ag, Au, etc.)	Antibacterial and catalytic activity	Improves fouling resistance	Biomedicine, water disinfection, sensors	[95,96]

**Table 7:** Performance Comparison of Composite Membranes for the Removal of Various Contaminants.

No.	Membrane Composition	Pollutant	Permeability (L·m^−2^·h^−1^·bar^−1^)	FRR (%)	Removal Efficiency (%)	Ref.
1	MXene/Ag_2_S/ cellulose	methylene blue	—	—	93.3	[110]
2	MXene (Ti_3_C_2_T_x_)/ cellulose	MG, BSA	—	67.3	99.49	[111]
3	Ti_3_C_2_T_x_ (MXene)/cellulose acetate	*E. coli*, *B. subtilis*	256.85	—	98	[112]
4	PSF/MXene	BSA	450	76.1	>90	[113]
5	PES/Zwitterion-MXene	BSA	480	94	90	[114]
6	MXene/ceramic substrate	Humic acid	331.9	—	86.5	[115]
7	PAN/CS/Fe_3_O_4_	Humic acid	25.5	—	96.5	[116]
8	PAN/ZnO-NPs	Mn^7+^	136.3	87.4	96.21	[117]
9	PES/mesoporous Si	Cd^2+^	~80	—	91	[118]
10	PES/mesoporous Si	Zn^2+^	20	—	94	[118]

## Data Availability

The original contributions presented in this study are included in the article. Further inquiries can be directed to the corresponding authors.

## Abbreviations

The following abbreviations are used in this manuscript:

CNC	Crystalline Nanocellulose
CNF	Cellulose Nanofibrils
BNC	Bacterial Nanocellulose
TEMPO	2,2,6,6-Tetramethylpiperidine-1-oxyl
MOF	Metal–Organic Framework
PES	Polyethersulfone
PANI	Polyaniline
PAN	Polyacrylonitrile
PVDF	Polyvinylidene Fluoride
CNT	Carbon Nanotubes
FRR	Flux Recovery Ratio
